# Mining sick: Creatively unsettling normative narratives about industry, environment, extraction, and the health geographies of rural, remote, northern, and Indigenous communities in British Columbia

**DOI:** 10.1111/cag.12660

**Published:** 2020-12-11

**Authors:** Terri‐Leigh Aldred, Charis Alderfer‐Mumma, Sarah de Leeuw, May Farrales, Margo Greenwood, Dawn Hoogeveen, Ryan O’Toole, Margot W. Parkes, Vanessa Sloan Morgan

**Affiliations:** ^1^ Carrier Sekani Family Services; ^2^ Department of Family Practice, Faculty of Medicine University of British Columbia; ^3^ Health Arts Research Centre University of Northern British Columbia; ^4^ Northern Medical Program University of Northern British Columbia; ^5^ Faculty of Medicine University of British Columbia; ^6^ Department of Geography Simon Fraser University; ^7^ Department of Gender, Sexuality and Women's Studies Simon Fraser University; ^8^ National Collaborating Centre for Indigenous Health; ^9^ First Nations Studies Program University of Northern British Columbia; ^10^ Environment Community Health Observatory Network University of Northern British Columbia; ^11^ School of Health Sciences University of Northern British Columbia; ^12^ Geography Program University of Northern British Columbia; ^13^ School of Environmental Planning University of Northern British Columbia; ^14^ School of Health Sciences University of Northern British Columbia

**Keywords:** pathologization, story, coloniality, extraction, health, territoire pathogène, discours, colonialité, extraction, santé

## Abstract

*Rural, remote, northern, and Indigenous communities on Turtle Island are routinely—as Cree Elder Willie Ermine says—pathologized. Social science and health scholarship, including scholarship by geographers, often constructs Indigenous human and physical geographies as unhealthy, diseased, vulnerable, and undergoing extraction. These constructions are not inaccurate: peoples and places beyond urban metropoles on Turtle Island live with higher burdens of poor health; Indigenous peoples face systemic violence and racism in colonial landscapes; rural, remote, northern, and Indigenous geographies are sites of industrial incursions; and many rural and remote geographies remain challenging for diverse Indigenous peoples. What, however, are the consequences of imagining and constructing people and places as “sick”? Constructions of “sick” geographies fulfill and extend settler (often European white) colonial narratives about othered geographies. Rural, remote, northern, and Indigenous geographies are discursively “mined” for narratives of sickness. This mining upholds a sense of health and wellness in southern, urban, Euro‐white‐settler imaginations. Drawing from multi‐year, relationship‐based, cross‐disciplinary qualitative community‐informed experiences, and anchored in feminist, anti‐colonial, and anti‐racist methodologies that guided creative and humanities‐informed stories, this paper concludes with different stories. It unsettles settler‐colonial powers reliant on constructing narratives about sickness in others and consequently reframes conversations about Indigenous well‐being and the environment*.

## Key Messages

- Myriad discourses converge to produce rural, remote, northern, and Indigenous geographies as pathologized spaces.
- Pathologizing rural, remote, northern, and Indigenous geographies leads to people and places being susceptible to discursive mining.
- New and different stories must be created and told about rural, remote, northern, and Indigenous geographies.

## Malade de l'industrie minière : la créativité troublante des discours normatifs sur l'industrie, l'environnement, l'extraction et la santé des communautés autochtones, nordiques et rurales en Colombie‐Britannique

*Les communautés autochtones, nordiques et rurales de Turtle Island sont, comme le dit l'aîné cri Willie Ermine, couramment considérées comme pathogènes. Le discours professoral en sciences sociales et en santé, y compris chez les géographes, conçoit souvent les géographies autochtones, tant humaines que physiques, comme étant malsaines, malades, vulnérables et soumises à l'extraction. Ces conceptions ne sont pas erronées: les gens et les endroits en dehors des agglomérations urbaines sur Turtle Island sont davantage exposés à un état de santé précaire, les Autochtones font face à une violence et un racisme systémique dans les milieux coloniaux, les géographies autochtones, nordiques et rurales sont le siège d'incursions industrielles et de nombreux contextes territoriaux ruraux et éloignés continuent de présenter un défi pour diverses populations autochtones. Toutefois, quelles sont les conséquences d'imaginer et de concevoir les gens et les endroits comme étant « malades »? Le concept de territoires « malades » favorise et consolide les récits coloniaux (souvent le blanc européen) sur l’état d'autres territoires, lesquels seraient dans une situation plus favorable. Les géographies autochtones, nordiques et rurales sont alors « minées » de manière discursive par les récits sur leur caractère pathogène. Ce minage soutient une impression de santé et de bien‐être dans l'imaginaire colonial, blanc européen et urbain des populations du sud. S'inspirant d'expériences communautaires qualitatives éclairées interdisciplinaires pluriannuelles axées sur les relations et ancrées dans les méthodologies féministes, anticoloniales et antiracistes qui ont guidé les discours créatifs inspirés par les sciences humaines, le présent texte se termine avec des récits différents. Ceux‐ci déstabilisent les pouvoirs coloniaux qui se fondent sur le concept de territoire pathogène et, par conséquent, ré‐interprètent les perceptions et les affirmations sur l'environnement et le bien‐être des Autochtones*.

## Introduction

We (us, the collection of authors) live, learn, work, love, mourn, and play across the messy complex geographies of so‐called northern British Columbia (BC). Northern BC is an area governed and stewarded for many thousands of years by 17 overlapping First Nations and, today, it is the home of close to 47,200 First Nation people representing 35.6% of the province's First Nations population (FNHA, n.d.). Thousands of Métis and non‐status Indigenous peoples share these same geographies. We all live, learn, work, love, mourn, and play across watersheds and mountain ranges that, if mapped in ways more consistent with settler‐imaginaries (as, for instance, a health authority or an electoral district or a postal code), is equal in size to France, that nation with such a long colonial relationship to the country now known to so many as Canada. We also all live, learn, work, love, mourn, and play together, every day, with an ever‐present “background noise” of news accounts about fracking, clear‐cut logging, open‐pit mining, oil and gas pipelines, forest fires, and salmon stock depletion. We live to the sounds of 18‐wheel trucks hauling logs from pine‐beetle‐killed trees mingling with broadcasts about missing and murdered Indigenous women and girls. We awake to social media pings about high crime rates and poor health outcomes, and we go to work amongst announcements about brand new research initiatives discovering new disparities in the communities we know as home.

While some of us are Indigenous to the lands of so‐called northern BC, with kinship lines that extend back into time immemorial, others of us are Indigenous from other parts of Turtle Island. Some of us are non‐Indigenous settler folx. Not all of us who are non‐Indigenous are white, which is important because we observe that, often, a moniker of “non‐Indigenous” or “settler” is automatically taken to mean white and of European descent. Which, to be clear, a number of us are. We are not all academics or researchers. We are varied in age as well as in sexual and gender orientation. We do not always agree on all things, but together we work on many things. In other words, we are a diverse group of people—a diversity reflective in many ways of the socio‐cultural and physical geographies that comprise the present‐day states of coloniality that are northern BC (and beyond).

We deeply believe that by writing this story *together*—as opposed, for instance, to the story being authored only by people Indigenous to northern BC—we are taking a geographically specific anti‐colonial stance against colonial modalities that insist on dualities and opposition and do not recognize complexity and slippery intersectionality (Tuck and Yang 2012). We are pushing forward with our emplaced and intimate knowledges, knowledges we believe arise from knowing, being, and loving in the places of northern BC. We hope our efforts are not read as exclusionary but, instead, as geographically specific, intimate writing arising in, from, and for a specific place (see Moss and Donovan 2017). In our effort of working together as diverse Indigenous peoples and diverse settlers, we are taking seriously the concepts of relationality, of being in relationship with “all our relations”—a teaching that insists not only upon the interconnectedness and responsibility of all humans to both each other and to non‐human beings, but also upon forging new ways of being together (Beniuk 2016). We are, we hope, embodying new modes of geographically anchored relationality for an as yet unknown future. Writing this, in addition to situating and positioning ourselves, is important conceptual work because, as many of us have written about elsewhere and as others have also observed, situating writers and researchers in reference to our work dispels myths about objective knowledge and thus disrupts historically masculinized Euro‐colonial conceits that research and writing are neutral acts documenting emotionless and placeless evidence (Haraway 1988; de Leeuw and Hunt 2018).

With all this said, however, it is important to understand nuanced gradations of power. For one, we understand that processes of power, like racialization in a white supremacist settler colonial capitalist context, give structure to the spectrum of who is considered human, who is considered barely human, and who is considered to lie outside the human, with uneven material effects (McKittrick 2006; King 2019). We write together, weary of such deep‐rooted and systemic gradations of power. We write together, cognizant that many of us are affiliated with institutions of coloniality. Many of us do work and study at a university that celebrates research chairs in climate change and water security, sponsored by industries responsible for the forceful dispossession of First Nations from northern lands/waters. For instance, on November 4, 2019 and while this paper was being written, the University of Northern British Columbia victoriously announced an NSERC/Rio Tinto Senior Industrial Research Chair in Climate Change and Water Security (UNBC 2019). Two weeks later, on November 18, 2019, Saik'uz and Stellat'en First Nations members provided their testimonies in the BC Supreme Court against Alcan's Rio Tinto. Those testimonies are an effort to hold the corporation accountable for the death and dispossession inflicted by the construction of the Kenney Dam in 1952, including the ongoing harm caused by Rio Tinto to the Nechako River and its invaluable fish populations (Saik'uz First Nation 2019; Wilson 2020). Court cases such as this are not unique between corporate interests and First Nations, in that the playing field is uneven and is often represented in court battles that ultimately come down to questions over jurisdiction and lands.

The messy complex geographies where we live, learn, work, love, mourn, and play are, in other words, deeply storied geographies—geographies storied, in part, by the stories we are part of creating. The stories, or the discursive narratives, are also powerfully formed through mass media and social media; government inquires and reports; social, environmental, science, and other research; policy documents, legal frameworks, and organizational initiatives; and health bulletins and advertisements. These stories are often negative, or what some of us have described as deficit‐based narratives (Greenwood et al. 2005; see also Tuck 2009; Wood et al. 2018).

In this paper, we conceptualize and critically interrogate those deficit‐based discursive narratives as a “story‐of‐sick.” Stories‐of‐sick, we suggest, form a broad understanding about the geographies of northern BC. That broad understanding, we go on to suggest, allows people not deeply imbedded or vested by kinship in or relationships with geographies of northern BC to understand themselves and their socio‐cultural and physical geographies in particular ways. The stories‐of‐sick, we contend, buttress and support in many people (with no lived or kinship connection to the geographies where we live, learn, work, love, mourn, and play) a kind of wellness narrative—a sense of being healthy. This wellness narrative and sense of well‐being is based on being at a conceptual and territorial distance from what is imagined to be, and storied as, the inherently lesser, if not downright diseased and toxic, geographies of rural, remote, northern, and Indigenous geographies. This contention is at the heart of our paper, a paper that is also engaging a larger theme of Indigenous peoples and the environment. We are fundamentally asking why, when we speak to (often non‐Indigenous, white) people about where we live in rural remote northern geographies, are we so often met with confused looks of semi‐disapproval? Why, when we look around the disciplines of (for instance) geography, medicine, or health sciences, are we inevitably met with research that seems to suggest the environments where we live are sick and toxic (deficit‐based) places? Why are our homescape environments so often rendered as ugly, exploited, unhealthy, damaged, emptied, or under threat? What, we wonder in this paper, are the implications of some environments being consistently *imagined as sick, geographically, ecologically, and with reference to health*? We are deeply inspired in our questions by other social scientists documenting ways that stories about vulnerability, and other acts of labelling circumpolar northern Indigenous communities, actually and deeply problematically work to reproduce and entrench pathologized misconceptions, especially in southern settler peoples’ imaginations (Haalboom and Natcher 2012).

This paper, in efforts to re‐story the geographies of northern BC, is itself and like so much scholarship, in many ways a story. While Indigenous peoples have an immeasurably long history of storywork and storytelling, the discipline of geography is also historied by story and, increasingly, is turning to stories as geographic method and methodology (Archibald 2008; de Leeuw et al. 2017; Nelson 2017; Justice 2018). We are interested, quite specifically, in linking the stories told about the terrains and health‐landscapes of rural, remote, northern, and Indigenous geographies to the histories and present‐day strategies of colonial projects and imaginings that, we argue, so often work to perpetuate a state of coloniality vested in pathologizing geographies rich with Indigenous presence, resistance, and re‐enlivening. Our theorizing and conceptualizing about pathologizing geographies, and how they serve a state of coloniality, is ultimately countered with a number of short vignettes and stories from ongoing community‐informed research and projects. These projects often use arts‐based methods and strength‐based tools that try, at least a little bit, to resist normative colonial extractive research tools. In other words, the stories we feature in this paper, the narrative empirics if you will, reach towards understandings about wellness and beauty and resilience and power and strength and potential in geographies that have never, ever, been relinquished by Indigenous peoples in what is now labelled, in settler colonial terms, as northern British Columbia on Turtle Island. These are geographies with stories ever‐unfolding.

We must pause for a moment to emphasize that the story we are telling in this paper, a story that ties together a number of stories, is a particular kind of story. Our paper/story is one that, in many ways, has been repeatedly told in multiple story forms by Indigenous academics and communities (e.g., Tuck 2009; Million 2014). These stories, told by Indigenous scholars, activists, and community members, often remain unheard by mostly white, settler academics living far away from the places about which often white, often settler, academics write and produce narratives and stories. It is important to recall Tanana Athabascan scholar Dian Million's (2014) assertion that “Indigenous concepts of how the world works, and how it came to be, can never be summarily dismissed. They work differently. Story has always been practical, strategic, and restorative. Story *is* Indigenous theory.” We, and particularly the Indigenous authors of our collective authorship group, emphasize that *Indigenous* stories are based on kinship ties, legal histories, and law, and being in relation to what many geographers deem “non‐human‐beings.” While highly acclaimed Indigenous authors and storytellers writing on the public record abound (see for instance Atleo 2004; Napoleon 2019), and while there are many powerful Indigenous storytellers writing for generations to come about kinship ties, legal histories, and law (see for instance Maracle 2015; Gray Smith 2017), we believe it is important to note that many Indigenous stories are not written down—they are not google‐able. These are stories held as life‐knowledge and ceremonies, stories shared and circulated internally to specific Indigenous kinship webs with care and in accordance to legal histories and protocol. These Indigenous stories are born of lands and waterways; they are stories that create definitions and subjectivities embedded in place, lands, water, spirituality, and being in relation. When settler‐scholars and storytellers seek to re‐tell stories of Indigenous places (for example Boyden 2005, 2013; see also Keeshig‐Tobias 1997), especially from faraway places lacking relational connectivity, stories of Indigenous origin can be viewed as somehow possessable by those to whom they do not belong. Or the stories can be erased altogether.

Stories as Indigenous theory, kinship, law, rights, and knowledges are *not* the kind of stories shared in this paper. Still, the story we are telling in this paper is intimately connected to lands, waters, health, well‐being, and each other. Indeed, as Cherokee scholar Thomas King (2003, 2) so eloquently asserts: “The truth about stories is that's all we are.” While geographers are not immune to stealing Indigenous knowledges and stories (Voosen 2016), it might be said that geographers, when undertaking scholarship about Indigenous geographies, are now more often creating, writing about, or employing stories in our research, as opposed to more actively or overtly stealing the stories of Indigenous peoples (e.g., Cameron 2012; de Leeuw et al. 2017). This is not to suggest that we are arguing against a need to critically engage, from an anti‐colonial perspective, the work of geographers. Instead, we are arguing for a deeper critical engagement with the discursive powers of our stories, even if they are not directly stolen, including the very modes used to tell stories and the themes around which stories operate. With this in mind, we hope our paper has wide reach for anticolonial geographers and health researchers, alongside other critical and social scientists. Ultimately, we see this paper as a reworking and rewriting of geographies pathologized by story. This reworking and rewriting may just result in the geographies we know as home being reimagined for healthier futures. From now onward.

## In relationship: Background and context

We believe all stories rest on, in some way, shape, or form, the stories of others. We are thus in relation to the many stories and storytellers all around us. Our ideas are anchored in the knowledge of others. Three basic premises undergird this paper. The first premise is that epistemologies (how we know what we know) and ontologies (the material “stuff” about which knowledge is known) are co‐constitutive. In other words, what is thought about and written about “stuff” (like rivers and rocks and bodies and diseases) is actually, in great part, what that “stuff” is. King (2003, 10) cautions, “You have to be careful with the stories you tell. And you have to watch out for the stories that you are told.” *What* stories are told about places and people, and *how* people tell each other those stories, matters. The second premise of this paper is that Indigenous knowledges, writings, voices, stories, and world views should *always be* the starting point of any work about or focusing on Indigenous peoples, communities, and geographies. There should furthermore be utility to research and writing being produced about Indigenous peoples, places, and communities. This starting point is in direct dialogue with a growing number of Indigenous scholars and activists who have started a global “citational challenge” (Tuck 2015; see also Ybarra 2019), which we are actively taking up in this paper, noting that knowledge must in part be evaluated by what it is rooted upon. In other words, knowledge and research about the rural, remote, northern and Indigenous geographies in BC that is not written by or anchored in, and full of citations and voices from, people in and of those places, with kinship ties to place, may not be research and knowledge with much utility to those whom the knowledge and research is about. The third premise underlying this paper is that damage is enacted when solutions about perceived challenges, produced by certain kinds of narratives, are offered from contexts that are tacitly taken as neutral and objective. Offering diagnoses and prescriptions to fix or heal ailing or unhealthy people, places, and ecologies, without carefully deconstructing the premise of health upon which those diagnoses and prescriptions are being made, is troublesome. Unangax scholar Eve Tuck (2009, 415) emphasizes that without foregrounding the larger context in which Indigenous communities are embedded, Nations become pathologized:

Without the context of racism and colonization, all we're left with is the damage, and this makes our stories vulnerable to pathologizing … evidence of ongoing colonization by research—absent a context in which we acknowledge that colonization—is relegated to our own bodies, our own families, our own social networks, our own leadership. After the research team leaves, after the town meeting, after the news cameras have gone away, all we are left with is the damage.

Tuck (2009, 417) contends that depathologizing studies—studies that in part foreground desire—can account for “the loss and despair, but also the hope, the visions, the wisdom of lived lives and communities” (see also Wood et al 2018).

The inherently critical, anti‐colonial, and feminist methodological framing of research and knowledge production of the three premises that underlay this paper are also deeply informed by Nishnaabeg scholar Leanne Betasamosake Simpson. Simpson (2017) has recently posed questions about the ways knowledge is made. Theory, most broadly and within the confines of Anglo‐Euro‐Western knowledge frameworks, refers to a system of ideas that explains something: theory is a set of principles upon which an activity or practice is premised. Simpson asks some poignant questions about theory as it pertains to Indigenous peoples. Her questions, at their core, are about human knowing and being in the world:

Where does this theory come from? What is the context? How was it generated? Who generated it? How is it useful in the context of my own [Indigenous] people? Can [we] use it in an ethical and appropriate way (my ethics and theirs) given the colonial context within which scholarship and publishing take place?

(Simpson 2017, 63)

Importantly, Simpson (2017, 63) demonstrates how her position and “Nishnaabeg grounded normativity provides the instigation for [the] wide intellectual engagement” required to ask such questions when theorizing in relation with other Nations and communities. Theories are not just the conceptual or methodological *underpinnings of research* but, instead, are entire structures of thoughts and beliefs that *define all aspects of the world*. To again echo Million (2014): “Story *is* Indigenous theory.” Theories must be applied in ethical and culturally attuned ways that recognize hegemonic systems of power—in ways that do not steal Indigenous stories (Keeshig‐Tobias 1997).

Taking to heart Simpson, Million, and Tuck's prompts, questions (and answers) about stories, environment, ecology, and health for Indigenous peoples means taking to heart the idea that theories systematize and structure understandings about each of these concepts. Health, Indigenous peoples (and settlers), and the ecologies in which life unfolds are impacted by the stories that circulate about them. The very separation of health from ecology is partly anchored in actions of theories and theoretical frameworks, in this case, colonial (mostly Euro‐white) ways of defining the world that conceptualize lands and waters—remember these are *Indigenous* lands and waters in northern BC—as somehow separate from health and well‐being. Katie Big‐Canoe and Chantelle Richmond (2014, 128) point out the falsity of this dichotomy, especially when exploring Anishinaabe youth perspectives on community well‐being; “herein,” they state, “is an important and often overlooked inter‐relatedness; the health of the land is inseparable from the health of those whose existence relies so indelibly on it.” Potawatomi scholar Robin Kimmerer (2013, 57) eloquently traces back how narratives we tell children at a young age either reproduce interconnectedness or not: “When we tell them [children] that the tree is not a *who*, but an *it*, we make that maple an object; we put a barrier between us, absolving ourselves of moral responsibility and opening the door to exploitation.”

With reference to the theme of Indigenous people's health and the environment (or ecologies and geographies), we now turn to various ways that knowledge is made about rural, remote, and Indigenous geographies in northern BC. To be clear, we are not seeking to tell Indigenous stories. We are, however, seeking to reveal how stories about the health of lands, waters, and peoples on unceded Indigenous territories in what is now referred to as northern BC create a narrative about peoples and places—what Ermine (2004) has termed the “pathologization” of communities. If human health and the lands and waters and beings of the non‐human world are understood, fundamentally, to be inseparable, we contend that the stories told about either or both are similarly inseparable. The more stories about underserviced, ruined, resource‐extracted, lands that are circulated about northern BC, the more people of northern BC are conflated with that place and understood as toxic: The more stories about the poor health of northern BC are circulated, the more lands and ecologies can be understood as deficient and toxic. Both these (co‐constitutive) narratives make conceptual and discursive space about a need to save, a need to fix, a drive to make healthy or (sometimes simultaneously) an ability to ignore that which is normalized as pathological and sick.

We must be clear about a few details. While all of northern BC, with some small exceptions where treaties have been negotiated (Nisga'a Nation and *Treaty 8*), are un‐treaty‐ed, unceded lands, not all Indigenous geographies in northern BC are rural or remote. While it is impossible to fully parse apart the northern from the rural from the Indigenous when considering geographies of BC beyond the 50^th^ parallel, it remains important to understand there are a series of intersectional and interconnecting considerations at play across northern BC and thus also at play as contexts to our conversations. These complex relationalities also mean we are acutely aware of gradations of power forming and informing the geographies about which we write and within which we live, learn, work, love, mourn, and play. While recognizing it is impossible to generalize our discussion across genders, abilities, class, sexual orientations, racializations, locations, stages of life, etcetera, we survey the influential literatures and research about Indigenous peoples, after which we move into scholarship and narratives about northern, rural, and remote geographies. We will chart broad patterns and ask: Who do these patterns serve and what structures of power do they uphold?

## Constructing illness: The making of Indigenous, northern, rural, and remote geographies

One of the most oft‐cited papers on the health status of Indigenous peoples in Canada, and amongst the first papers that come up if you do a “Google Scholar” search for Indigenous health in Canada, is Naomi Adelson's (2005) now almost‐canonical paper “The embodiment of inequity: Health disparities in Aboriginal Canada.” This was indeed a groundbreaking piece of research, one that cemented for most health researchers an understanding that Indigenous peoples in Canada live with a disproportionate burden of ill health and social suffering as compared with non‐Indigenous Canadians (Adelson 2005). There are other landmark studies about Indigenous peoples’ health, both in Canada and around the world. These studies have put on the public record, time and time again, that Indigenous peoples, compared with non‐Indigenous peoples, have much shorter lifespans and face higher rates of chronic and infectious diseases as well as higher rates of suicide and domestic violence (Frohlich et al. 2006; Health Council of Canada 2012). These disparities are by no means “even”: Indigenous women, queer or Two‐Spirit Indigenous youth, and Indigenous people living in poverty or in isolated, underserved communities have even greater burdens of poor health than other Indigenous individuals. The annual BC Provincial Health Officer's report documents reams and reams of challenges facing Indigenous peoples in the Northern Health Authority relative to other provincial health jurisdictions, including increased rates of co‐morbidities, higher rates of tobacco and alcohol consumption, and high rates of HIV/AIDS (to name but a few) (Office of the Provincial Health Officer 2019). Increasingly, and with a growing discourse about the social determinants of health, it is well‐evidenced that Indigenous peoples’ health is deeply determined by historic and contemporary colonial violence, by an ever‐present state of coloniality, and by systemic anti‐Indigenous racism (Czyzewski 2011). These evidences often rest on deeply upsetting cases of Indigenous patients left to die in emergency wards or suicide epidemics amongst Indigenous communities facing substandard servicing (Allan and Smylie 2015).

This is not to mention the racism Indigenous people face in accessing health care in hospitals in semi‐urban centres—well‐documented in places such as, for example, Williams Lake, Thunder Bay, and Prince George (Allan and Smylie 2015). Much of the research and literature about urban Indigenous peoples and communities, both in popular media and academic publications, focus on socio‐cultural and economic exploitation, while research and literatures on “reserve” spaces similarly focus on violence, poverty, and deprivation (Hunt 2014). Indeed, as Kwagiulth geographer scholar and activist Sarah Hunt (Tłaliłila'ogwa) and co‐authors point out, there is a tacit linking of violence and Indigenous peoples and communities, a naturalization of an imagined state that allows the colonial state to ignore needs, voices, laws, and protocols of Indigenous people because violence and pathology are imagined as *normal* (Hunt 2014; Holmes et al. 2015; Hunt and Holmes 2015). Geographer Michael Fabris (2016), from the Piikani Nation, reveals how attempts to privatize reserve lands as fee simple is an act of primitive accumulation. Illuminating how discourse and imaginings about faraway “reserve lands” allow for imaginations to become grounded, Fabris connects federal efforts of transforming reserve lands into fee simple systems with discourse and narratives that suggest the need to “fix” reserves. Such transformations mirror other and ongoing settler colonial assimilation attempts, often linked with policies anchored in myopic imaginations of “saving” Indigenous peoples and lands, including, for instance, Canada's 1969 *White Paper* (Fabris 2016). Work like that of Hunt, Fabris, and others counter these deficit‐based imaginations that focus—sometimes in spectacular ways (see Baloy 2016 and Daigle 2019 on the spectacle of reconciliation)—on poverty, violence, and indeed sickness as justifications for assimilation, reconciliation, and erasure.

We are not suggesting that studies and bodies of evidence about challenges in northern, rural, or Indigenous places are untrue. A number of us have, in fact, authored similarly‐toned health research, observing that Indigenous peoples are, indeed, people of poorer health as compared to non‐Indigenous peoples in Canada (de Leeuw et al. 2012; Greenwood et al. 2015). We are also not suggesting that such research should not exist; we are, however, suggesting that these stories tell, and produce, a certain picture of Indigenous people's health in Canada. It is a picture of sickness and damage, a picture of despair and lack and illness. If one were to look at different scales, or change the aperture of what was being focused upon, might a different story emerge? For instance, a number of years ago two very influential researchers authored a disruptive and transformative study about suicidal ideations in First Nations communities. The researchers observed that while aggregated data told a story of a suicide epidemic in Canada, that story missed an entirely different narrative if the data were disaggregated and, instead, individual communities (many of which had lower rates of youth suicide than the national average) were looked at (Chandler and LaLonde 1998). What could be learned, asked the researchers, if smaller‐scale successes and strengths were not masked within a more totalizing narrative? In other words, the *way* a story is told can produce widely different understandings about the same topic.

It is now well‐critiqued, but worth repeating, that the normative way that Canada tells its story of nation‐building, and the story that the so‐called province of British Columbia tells of itself, is a particular kind of a narrative that posits the white heteropatriarchal masculine subject as the builder of nations, cities, industry, and even “modernity.” There is a longer genealogy of the making of what Black scholar Tiffany King (2019) might call this “conquistador human,” but what's worth pointing out here is the series of disavowals, violent assimilations, and erasures of Indigenous, Black, and communities of colour necessary for this totalizing narrative to gain coherence. The totalizing narrative of rural and remote geographies as always‐and‐already backward and sick is inherently a racial and heteropatriarchal one.

In addition to the plethora of literatures, evidences, and research about the diminished status of Indigenous peoples’ health, there is also a substantial and growing body of work that documents how the ecologies, environments, and geographies inhabited by Indigenous peoples are also toxic places and spaces of exploitation, extraction, and suffering. Mass and popular media often focus on the lack of clean water in many First Nations reserves, on pipeline incursions, or on violence and despair in Indigenous families and homes marred by poverty unaddressed by vast wealth in the resource sector. These narratives have embodied consequences. The forming of pejorative narratives can, for instance, form pejorative understandings about Indigenous peoples when they seek care in the healthcare system, resulting in targeted racism (Browne and Varcoe 2006). Indeed, recent research about Canada's media coverage of Indigenous peoples found the vast majority of content focused on aberrations and illnesses (Johnson 2018). In ongoing efforts to critically understand environmental and ecological carnage, and often within research and writings about the Anthropocene, an increasing number of scholars and activists are documenting the violence unfolding on unceded territories of Indigenous peoples around the world or within lands, waters, and ecosystems stewarded by Indigenous peoples (see for instance Davis and Todd 2017; Whyte 2017). Writing on the Anthropocene and/or anthropogenic causes of climate change has itself perpetuated violence against Indigenous peoples and lands. For instance, Potawatomi scholar and activist Kyle Whyte (2017) recounts that anthropogenic climate change must be approached with the understanding that “Colonially‐driven environmental change destroyed ecosystems on which Indigenous peoples relied, boxed Indigenous peoples into small reservations that were fractions of their original territories, or simply displaced Indigenous peoples from their homelands to new ecosystems.”

“An analysis of Indigenous climate vulnerability,” Whyte (2017) contends, “cannot occur in the absence of the history and present practices of colonialism and capitalism in Indigenous homelands.” Whyte insists on making visible links between colonialism, capitalism, and anthropogenic climate change—connections that are often erased in mainstream discourse and dialogue on resilience and adaptation, health, and well‐being; he thus refuses to dislocate land‐based violence with that experienced by peoples embedded in place.

Whyte is not alone in his arguments. Heather Davis and Métis/Otipemisiw scholar Zoe Todd (2017, 763) point to similar concerns, asserting that:

By linking the Anthropocene with colonization, it draws attention to the violence at its core, and calls for the consideration of Indigenous philosophies and processes of Indigenous self‐governance as a necessary political corrective, alongside the self‐determination of other communities and societies violently impacted by the white supremacist, colonial, and capitalist logics instantiated in the origins of the Anthropocene.

Davis and Todd (2017) align with Whyte (2017), who himself maintains that “anthropogenic climate change is an intensified repetition of anthropogenic environmental change inflicted on Indigenous peoples via colonial practices that facilitated capitalist industrial expansion.” Failing to recognize these connections means failing to reflect on the impact that dynamics dictating relationships to land have on health itself; doing so dislocates health from place, decontextualizes well‐being from being‐in‐relation, and allows more easily for lands and peoples to be labelled as “sick.”

There are also works within the domain of waste and toxicity, such as *Wastelanding* by Traci Voyles (2015), that point out and criticize narratives of racialized environments as marginal, worthless, and pollutable “wastelands.” Voyles (2015, 9) argues that wasteland “has been a key and unexplored component of environmental racism.” Further to these significant contributions concerning violence, land, the environment, and settler colonialism, Voyles makes a feminist move to scale down from macro‐geographies of territory to micro‐geographies of the body. She argues that “colonial epistemologies do not just look on deserts as wastelands but that wastelands of many kinds are constituted through racial and spatial politics that render certain bodies and landscapes pollutable” (Voyles 2015, 10). This dovetails with our critique of toxicity and pathology, spurring our own call to move away from conceptualizations about polluted lands (and bodies) to an approach grounded in critical analyses of settler colonial dispossession. This is not to say critical analyses of settler colonial dispossession are without flaw. We acknowledge that much of that analysis is penned principally by non‐Indigenous (often white) geographers and non‐Indigenous anthropologists. We agree that such analytical lenses often emerge out of colonial literary and research traditions that construct Indigenous subjectivity in ways that do not always take on white supremacy, while at the same time *positing* an anti‐colonial lens.

Our intent is not to wholesale refute or refuse these readings of environmental violence and the bodily impacts. Instead, we are interested in charting and unpacking conceptual lenses that, we argue, ultimately begin with deficit‐oriented thinking. In doing so, we aim to bring attention to notions of toxic pathology, or what we refer to as “stories‐of‐sick.” Authoring stories‐of‐sick allows for those *outside* the toxicity about which they are writing and conceptualizing to mine—to extract—a sense of self that is healthy and distanced from the toxicity they imagine and construct. Examples of polluted resource‐extractive environments are plenty in geography, anthropology, and environmental studies. In shifting discussions *away from toxic environments*, like mining waste or pipeline incursions or underserviced boom and bust economies (Hanlon and Halseth 2005; Keeling and Sandlos 2009; McCreary and Milligan 2014, 2018), to a *critique of toxic narratives themselves*, we move to centralize understandings of health within revitalized and resurgent environments and rural, remote, northern, and Indigenous landscapes (Daigle 2018). Too often geographers and other social and health scientists forming narratives about rural, remote, northern, or Indigenous geographies, some of us included, either separate the intimate domestic and embodied from the ecological and natural environment or conflate all scales (from body to territories of water and land) into an undifferentiated mushy conglomerate of sick and extracted from. This, we contend, must be countered.

## Coloniality and pathologization: What stories‐of‐sick do for those who want to feel healthy

Opening this paper, we highlighted tendencies to pen northern locales as faraway places—places deeply disrupted and challenged, yet somehow and simultaneously removed and othered to those penning the stories about rural, remote, northern, and Indigenous geographies. We contend that such distancing—which is both physical and imaginary, both grounded and conceptual—allows a too‐often southern metropolitan (often white masculinized) narrative to be written. This narrative is one that we argue allows for pathologized narratives to be re‐inscribed—re‐storied onto northern places. Those writing about and busily creating this distance do so both literally and morally. Indeed, re‐inscribing distance through not seeing themselves/ourselves as culprits of the colonial present is deeply characteristic of settler colonialism, and of the extractive relations that are perceived as the sole economic drivers of northern BC. Speaking to the settler colonial contexts in the United States, Whyte (2018) elaborates on settler futurity that helps to guide our thinking here:

These ancestors [French, British, and U.S. colonists and settlers] would have relished the very idea that they could advance whatever business interests they wanted without facing threats of empowered resistance and diplomacy. They would have delighted in the idea that their legal orders would not have had to bend to or accommodate Indigenous legal orders. Many of the ancestors of today's allies designed the worlds we live in today to fulfil their fantasies of the future. Today's worlds, such as those of U.S. settler colonialism in North America, were constructed to provide privileges to their descendants. They were gifts of a troubling sort.

The “gifts” provided by settler ancestors—ancestors to many of the authors of this paper—make way for lands, lives, and territories to be re‐inscribed. These gifts also include a sense of what constitutes “good” health, including how humans should relate to lands and what decisions must be made that concern health. The gifts about which Whyte writes ultimately narrow and restrict expressions of well‐being, rendering both giver and receiver of said gifts as ultimately both unrecognizable and unaccountable. As long as (mostly white) settler‐geographers write about rural, remote, northern, and Indigenous geographies (in, for instance, northern BC) as toxic unhealthy places—both ecologically and socio‐culturally—they never need to see themselves/ourselves as part of the issue, as tied to a “gift” that constantly allows for the gift of distance and neutrality to be made invisible. As inheritors of the gift, we're/they're always more likely to be healthy, to be not(as)toxic.

Such distancing is necessary for colonial logics; it also buttresses racial capitalism at work in our communities. Writing from settler‐colonial, capitalist United States, Laura Pulido shows how the targeted geographical practices of waste are located in the very logic of racial capitalism. Pulido (2017, 527) asserts, “if racism is continually creating differential value, it is only logical that capital (and other nondemocratic economic systems) would incorporate this uneven geography of value into its calculus.” Pulido argues that, ultimately, racially devalued bodies (and we would suggest racialized and Indigenized spaces and places) act as “sinks” where the pollution and excess of industrial activities and capitalist lifestyles are dumped. Again, this dumping is both material and discursive, both physical and imaginary, and includes stories we tell ourselves and tales we extract and extend.

Million (2013) explores how health, healing, and trauma are orchestrated by the settler state. She describes how a “wounded” Indigenous subjectivity is “an agonistic site in the affective and moral power relations wherein Indigenous peoples articulate their self‐determination within a neoliberal Canada” (Million 2013, 6). Through these narratives, Million outlines how the state—and researchers—have flooded definitions of what constitutes healing, health, and trauma, and offloaded these definitions as “subjectivities.” Elsewhere, Million picks up this thread to enliven an alternate narrative, asserting how self‐determined story *is* Indigenous theory, and how the power of Indigenous narratives creates meaning by and for peoples in self‐determined ways:

Narratives seek inclusion; they seek the nooks and crannies of experiences filling cracks and restoring order. Narratives lay boundaries. Narratives give orphans homes. Narratives both make links and are the links that have been made. Narratives are our desire to link one paradigmatic will to knowledge to discursive and material projects that have consequences. Narratives serve the same function as any theory, in that they are practical vision….The felt experience of Indigenous experience in these Americas is in our narratives and that has made them almost unrecognizable to a Western scholarship that imagines itself objective.

(Million 2013, 35)

To what end—and for what purposes—do often white‐geographical narratives that bring the “gift” of good health inscribe distancing and re‐inscribe sickness? Can Western scholarship unpack its conception and distancing of “healthy” when health in faraway places is steeped in colonial narratives? We are inspired by Million's words here to critically look at how our research props up narratives of “healthy.” Indeed, following Million, we now shift to explore how communities and health care practitioners from Indigenous communities in northern BC are re‐narrating health and healing to re‐story northern geographies.

## (En)countering the story: How we're pretty healthy

Close your eyes. Imagine gatherings around kitchen tables. There are cans of salmon and slabs of fresh halibut. There is laughter and at least four generations of family. People are telling stories and laughing. There is a lot of laughter.

Close your eyes again and try to imagine something else. Imagine two booklets, each one only slightly larger than the palm of your hand. The cover of one is a cacophony of red and black, soft, tanned, leather‐clad feet carefully stepping forward toward you from a frame of swishing white tassels and the telltale ovoids of northwest coast Indigenous iconography (a language unto itself). The cover of the other booklet is a silvered, cedar totem face, a wise but somehow utterly inviting gaze, calling out to any reader. The booklets are offered as gifts to future and current health care providers in northern BC, with the caveat of “don't get discouraged, we are here to help you [learn our language]” (Indigenous Health Improvement Committee 2019). The booklets are full of Gitxsan words that would allow any health care provider to know the human body anatomically in Gitxsan, not to mention words of greeting and diagnoses. *Luu amhl goodi'y win gya'an* allows a nurse, social worker, or doctor to say, “I am happy to see you” to a patient while *Hindahl wila win?* asks, “How are you?” The booklets also contain words for places and feelings, for spiritual prayers and offerings—as resources, the booklets inherently link health to place, language, and culture. They are also testaments of strength. Of living health. The booklets were the dreams and work of the Northwest Aboriginal/Indigenous Health Improvement Committee, itself an outcome of a health authority (Northern Health) that was the first in Canada to appoint an Indigenous woman as Vice President of Indigenous Health. When the booklets were launched in 2017 and 2018, a gathering of more than 150 people from across northern BC laughed in unison as we learned the Gitxsan word for “butt.” Say it loudly and together, a Gitxsan Elder encouraged us: *Ts'im bok’! Ts'im bok’!* Humour and laughter abounded, both felt to be medicines unto themselves.

Now imagine more than 20 Indigenous youth between 10 and 14 years old, from more than 10 First Nations across northern BC, lining up to practise resuscitation on SymDolls in the region's largest hospital—each of the dolls is a state‐of‐the‐art piece of technology worth more than $100,000. The youth arrived at the SymLab after completing a land‐based medicine walk led by a Hereditary Chief and Elder, after making a series of short stop‐motion animation films later posted on YouTube, after drumming and singing and sharing stories about health as connected to land and water, and after learning how to gut and cure a salmon. What you are imagining is northern BC's first Indigenous Summer Science Camp, a partnership between the Northern Region of the First Nations Health Authority, the Northern Health Authority, and the Northern Medical Program's Health Arts Research Centre (HARC 2020).

Stretch your imagination a little further. Conjure 50 youth (many Indigenous, many racialized as not‐white) from northern BC joined by a Lheidli T'enneh Nation singer/songwriter, a Filipino hip hop artist and mental health advocate, and a kendama performer extraordinaire. Conjure an evening of performances about healthy environments and communities, with youth taking part in a friendship dance, trying their hands at the kendama, and collectively keeping the beat for performers during two hours of events all speaking about relationships to land and water during times of resource extraction. Youth wrote poems and identified what they saw as the strengths, challenges, and priorities for their community through hands‐on exercises and sharing circles (Sloan Morgan and Farrales 2020).

How about a few other stories to consider? Northern BC is home to Canada's longest‐standing Indigenous health research and knowledge translation institute—the National Collaborating Centre for Indigenous Health—which addresses First Nations, Inuit, and Métis peoples’ well‐being from coast to coast to coast and is the only institute of its kind in the country. Or, what about the successes of Indigenous physicians, nurses, and other health care providers either returning to their home territories in northern BC or moving to territories new to them in order to provide Indigenized care and combat systemic anti‐Indigenous racism in the health care system? A return to home is certainly part of productive efforts by doctors (including those who co‐author this paper) who are working with local Divisions of Family Practice to undertake everything from reconciliation training in hospital and emergency wards to running arts‐based health groups in remote First Nations who do not have a dedicated physician. Or what about the more than 50 undergraduate medical students training in northern BC, who, as part of the Truth and Reconciliation Committee's Calls to Action and commitments to the United Nations Declaration on the Rights of Indigenous Peoples, are annually placed in very remote northern First Nations for extended experiential community‐based learning programing? What about stories as counter‐narratives, stories that are the sharing of Indigenous and settler engagements with watersheds in northern BC, in ways that highlight ongoing relationships and collective efforts focused on connections of land, water, health, and well‐being (Gislason et al. 2018)? Where are these stories and narratives amidst tales of murdered and missing women and girls, resource extraction, and poor health outcomes? What happens when these stories are integrated into broader narratives and discourses about northern BC—and not just as “local” tales that provide small, accessorized bits of colour to larger and “more important” panaceas about longitudinal truths, epidemiolocal trends, historical transformations, or corporate extractive hegemony but, instead, as indicative of lived, grounded, everyday realities? What would happen if people with the power to write about rural, remote, northern, and Indigenous peoples and places did so not from a distance, but from here? What change to stories would the world see if we—those of us with the privilege to write, publish, and disseminate stories—all lived and laughed alongside Gitxsan Elders sharing words that connect our bodies with language, land, water, and culture?

## Together and well: Some conclusions about how to not pathologize rural, remote, northern, and Indigenous geographies—in BC and beyond

We suspect it is a certain kind of colonial corporate conceit to suggest clear answers and tidy conclusions. Let it be known and clearly stated, then, that we remain firmly rooted in a sense of uncertainty and humble confusion. We do not want stories about the challenges and difficulties and illnesses of rural, remote, northern, and Indigenous geographies to simply cease. Rather, what we are calling for is a surfacing, an awareness of, and a realization that distancing one's self, through critique and counter‐narrative constructions of people and places (geographies), might ultimately be re‐inscribing of (especially white urban) coloniality. This distancing, this constant attention to sickness and pathology and toxicity and extraction, never demands that those (of us) who are busily producing distance and finding problems actively see and understand them/ourselves as culprits in the colonial present. Instead, we get to be objective, neutral, *distanced observers* of the problems, here to diagnose and offer solutions. In other words, as long as (mostly white) geographer settlers write about rural, remote, northern, and Indigenous geographies (in for instance northern BC) as toxic unhealthy places—both ecologically and socio‐culturally—we (mostly white settler geographers) never need to *really* see themselves/ourselves as actively and right now part of the issue. As we've pointed out earlier in this paper, we're/they're always healthy and not (as) toxic. We're able to distance ourselves from a colonial present that, of course, we are deeply and intimately implicated within and complicit in perpetuating.

What might be needed, ultimately, is an intensely reflective effort at balance, at an always unsettling of how and what we write. Different stories need to be told. Stories need to be told different ways. Different people need to tell stories. New narratives need to be imagined and given space. Perhaps the best place to start is with the land and the water: they are never static, never unchanging. If health and well‐being are linked to the strength of ecology and environment, if medicine is story and story always rests in place, perhaps it is time to speak and learn stories of and in place. Perhaps it is the time (and place) to stop mining lands and waters and ecologies and environments of rural, remote, northern, and Indigenous geographies for stories of pathology. Perhaps, instead, new stories can be centred, stories focused on geographies of health and well‐being.

## References

[cag12660-bib-0001] Adelson, N. 2005. The embodiment of inequity: Health disparities in Aboriginal Canada. Canadian Journal of Public Health 96(2): S45–S61. 10.1007/BF03403702 PMC6975716 16078555

[cag12660-bib-0002] Allan, B. , and J. Smylie . 2015. First peoples, second class treatment: The role of racism in the health and well‐being of Indigenous peoples in Canada. Toronto, ON: The Wellesley Institute. https://www.wellesleyinstitute.com/wp-content/uploads/2015/02/Full-Report-FPSCT-Updated.pdf.

[cag12660-bib-0004] Archibald, J. A. 2008. Indigenous storywork: Educating the heart, mind, body, and spirit. Vancouver, BC: UBC Press.

[cag12660-bib-0003] Atleo, R. 2004. Tsawalk: A Nuu‐chah‐nulth worldview. Vancouver, BC: UBC Press.

[cag12660-bib-0007] Baloy, N. 2016. Spectacles and spectres: Settler colonial spaces in Vancouver. Settler Colonial Studies 6(3): 209–234.

[cag12660-bib-0005] Beniuk, J. 2016. All my relations: Reclaiming the stories of our Indigenous grandmothers. Atlantis: Critical Studies in Gender, Culture & Social Justice 37(2): 161–172.

[cag12660-bib-0006] Big‐Canoe, K. , and C. A. M. Richmond . 2014. Anishinabe youth perceptions about community health: Toward environmental repossession. Health & Place 26(March): 127–135. 10.1016/j.healthplace.2013.12.013. 24440804

[cag12660-bib-0008] Boyden, J. 2005. Three day road. Toronto, ON: Penguin Canada.

[cag12660-bib-0009] Boyden, J. . 2013. The orenda. Toronto, ON: Penguin Canada.

[cag12660-bib-0010] Browne, A. J. , and C. Varcoe . 2006. Critical cultural perspectives and health care involving Aboriginal peoples. Contemporary Nurse 22(2): 155–168. 17026422 10.5172/conu.2006.22.2.155

[cag12660-bib-0011] Cameron, E. 2012. New geographies of story and storytelling. Progress in Human Geography 36(5): 573–592. 10.1177/0309132511435000.

[cag12660-bib-0012] Chandler, M. J. , and C. Lalonde . 1998. Cultural continuity as a hedge against suicide in Canada's First Nations. Transcultural Psychiatry 35(2): 191–219.

[cag12660-bib-0013] Czyzewski, K. 2011. Colonialism as a broader social determinant of health. The International Indigenous Policy Journal 2(5): 1–14.

[cag12660-bib-0015] Daigle, M. 2018. Resurging through Kishiichiwan: The spatial politics of Indigenous water relations. Decolonization: Indigeneity, Education & Society 7(1): 159–172.

[cag12660-bib-0016] Daigle, M. . 2019. The spectacle of reconciliation: On (the) unsettling responsibilities to Indigenous peoples in the academy. Environment and Planning D: Society and Space 37(4): 703–721.

[cag12660-bib-0014] Davis, H. , and Z. Todd . 2017. On the importance of a date, or, decolonizing the Anthropocene. ACME: An International E‐Journal for Critical Geographies 16(4): 761–780.

[cag12660-bib-0017] de Leeuw, S. , and S. E. Hunt . 2018. Unsettling decolonizing geographies. Geography Compass 12(7). 10.1111/gec3.12376.

[cag12660-bib-0018] de Leeuw, S. , S. Maurice , T. Holyk , M. Greenwood , and W. Adam . 2012. With reserves: Colonial geographies and First Nations health. Annals of the Association of American Geographers 102(5): 904–911.

[cag12660-bib-0019] de Leeuw, S. , M. W. Parkes , V. Sloan Morgan , J. Christensen , N. Lindsay , K. Mitchell‐Foster , and J. Russell Jozkow . 2017. Going unscripted: A call to critically engage storytelling methods and methodologies in geography and the medical‐health sciences. The Canadian Geographer. 10.1111/cag.12337.

[cag12660-bib-0020] Ermine, W. , R. Sinclair , and B. Jeffery . 2004. *The ethics of research involving Indigenous Peoples: Report of the Indigenous People's Health Research Centre to the Interagency Advisory Panel on Research Ethics*. http://drc.usask.ca/projects/legal_aid/file/resource385-2c4c0417.pdf.

[cag12660-bib-0021] Fabris, M. P. C. 2016. Beyond the new Dawes Act: A critique of the First Nations Property Ownership Act. MA Thesis, Faculty of Graduate and Postdoctoral Studies, University of British Columbia. https://open.library.ubc.ca/cIRcle/collections/ubctheses/24/items/1.0308596.

[cag12660-bib-0022] FNHA (First Nations Health Authority) . n.d. *Northern*. http://www.fnha.ca/about/regions/north.

[cag12660-bib-0023] Frohlich, K. L. , N. Ross , and C. Richmond . 2006. Health disparities in Canada today. Health Policy 79(2–2): 132–143. 16519957 10.1016/j.healthpol.2005.12.010

[cag12660-bib-0024] Gislason, M. K. , V. Sloan Morgan , K. Mitchell‐Foster , and M. W. Parkes . 2018. Voices from the landscape: Storytelling as emergent counter‐narratives and collective action from northern BC watersheds. Health & Place 54: 191–199. 10.1016/j.healthplace.2018.08.024. 30321859

[cag12660-bib-0025] Gray Smith, M. 2017. Speaking our truth: A journey of reconciliation. Victoria, BC: Orca Book Publishers.

[cag12660-bib-0026] Greenwood, M. , S. de Leeuw , N. M. Lindsay , and C. Reading , eds. 2015. Determinants of Indigenous Peoples’ Health. Toronto, ON: Canadian Scholars’ Press.

[cag12660-bib-0027] Greenwood, M. , S. Tagalik , and S. de Leeuw . 2005. Beyond deficit: Exploring capacity building in Northern and Indigenous youth communities through strength‐based approaches. In The youth of British Columbia: Their past and their future, ed. R. Tonkin and L. Foster . Victoria, BC: Western Geographical Press, 175–188.

[cag12660-bib-0028] Haalboom, B. , and D. C. Natcher . 2012. The power and peril of “vulnerability”: Approaching community labels with caution in climate change research. Arctic 65(3): 319–327.

[cag12660-bib-0029] Hanlon, N. , and G. Halseth . 2005. The greying of resource communities in northern British Columbia: Implications for health care delivery in already‐underserviced communities. Canadian Geographer/Le Géographe canadien 49(1): 1–24.

[cag12660-bib-0030] Haraway, D. 1988. Situated knowledges: The science question in feminism and the privilege of partial perspective. Feminist Studies 14(3): 575–599.

[cag12660-bib-0033] HARC (Health Arts and Research Centre) . 2020. *Final Report and Video: Northern BC Indigenous Youth Science and Health Summer Camp*. http://healtharts.ca/final-report-video-northern-bc-indigenous-youth-science-and-health-summer-camp/.

[cag12660-bib-0031] Health Council of Canada . 2012. *Empathy, dignity, and respect: Creating cultural safety for Aboriginal people in urban health care*. Toronto, ON: Health Council of Canada. https://healthcouncilcanada.ca/files/Aboriginal_Report_EN_web_final.pdf.

[cag12660-bib-0032] Holmes, C. , S. Hunt , and A. Piedalue . 2015. Violence, colonialism and space: Towards a decolonizing dialogue. ACME: An International Journal for Critical Geographies 14(2): 539–570.

[cag12660-bib-0034] Hunt, S. E. 2014. Witnessing the colonialscape: Lighting the intimate fires of Indigenous legal pluralism. PhD Dissertation, Environment: Department of Geography, Simon Fraser University.

[cag12660-bib-0035] Hunt, S. , and C. Holmes . 2015. Everyday decolonization: Living a decolonizing queer politics. Journal of Lesbian Studies 19(2): 154–172. 25760993 10.1080/10894160.2015.970975

[cag12660-bib-0036] Indigenous Health Improvement Committee . 2019. Gitxsan Phrase Book. Smithers, BC: Northwest East (Smithers and Area) Indigenous Health Improvement Committee. https://www.indigenoushealthnh.ca/sites/default/files/Gitxan%20Phrase%20Booklet%20Volume%202.pdf.

[cag12660-bib-0037] Johnson, M. 2018. Stories we tell about “others”: Pathologizing discourses in mainstream media and their role as a distal determinant of Indigenous peoples’ health. MA Thesis, Interdisciplinary Studies, University of Northern British Columbia. 10.24124/2018/58839.

[cag12660-bib-0038] Justice, D. H. 2018. Why Indigenous literatures matter. Waterloo, ON: Wilfrid Laurier University Press.

[cag12660-bib-0039] Keeling, A. , and J. Sandlos . 2009. Environmental justice goes underground? Historical notes from Canada's northern mining frontier. Environmental Justice 2(3): 117–125.

[cag12660-bib-0040] Keeshig‐Tobias, L. 1997. Stop stealing Native stories. In Borrowed power: Essays on cultural appropriation, ed. B. Ziff and P. Rao . New Brunswick, NJ: Rutgers University Press, 71–73.

[cag12660-bib-0041] Kimmerer, R. W. 2013. Braiding sweetgrass. Minneapolis, MN: Milkweed Editions.

[cag12660-bib-0042] King, T. 2003. The truth about stories: A Native narrative. Toronto, ON: House of Anansi.

[cag12660-bib-0043] King, T. L. 2019. The Black shoals: Offshore formations of Black and Native Studies. Durham, NC: Duke University Press.

[cag12660-bib-0044] Maracle, L. 2015. Memory serves: Oratories, ed. S. Kamboureli . Edmonton, AB: NeWest Press.

[cag12660-bib-0045] McCreary, T. A. , and R. A. Milligan . 2014. Pipelines, permits, and protests: Carrier Sekani encounters with the Enbridge Northern Gateway project. cultural geographies 21(1): 115–129.

[cag12660-bib-0046] McCreary, T. A. , and R. A. Milligan . 2018. The limits of liberal recognition: Racial capitalism, settler colonialism, and environmental governance in Vancouver and Atlanta. Antipode 1–21. 10.1111/anti.12465.

[cag12660-bib-0047] McKittrick, K. 2006. Demonic grounds: Black women and the cartographies of struggle. Minneapolis, MN: University of Minnesota Press.

[cag12660-bib-0048] Million, D. 2013. Therapeutic nations: Healing in an age of Indigenous human rights. Phoenix, AZ: University of Arizona Press.

[cag12660-bib-0049] Million, D. . 2014. There is a river in me: Theory from life. In Theorizing Native Studies, ed. A. Simpson and A. Smith . Durham, NC: Duke University Press, 31–42. 10.1215/9780822376613-002.

[cag12660-bib-0050] Moss, P. , and C. Donovan , eds. 2017 Writing intimacy into feminist geography. Abingdon, UK: Taylor & Francis.

[cag12660-bib-0051] Napoleon, V. 2019. Did I break it? Recording Indigenous (customary) law. Potchefstroom Electronic Law Journal 22: 1–35.

[cag12660-bib-0052] Nelson, M. K. 2017. Getting dirty: The eco‐eroticism of women in Indigenous oral literatures. In Critically sovereign, ed. J. Barker . Durham, NC: Duke University Press, 229–260.

[cag12660-bib-0053] Office of the Provincial Health Officer . 2019. *Taking the pulse of the population: An update on the health of British Columbians*. https://www2.gov.bc.ca/gov/content/health/about-bc-s-health-care-system/office-of-the-provincial-health-officer/reports-publications/annual-reports.

[cag12660-bib-0054] Pulido, L. 2017. Geographies of race and ethnicity II: Environmental racism, racial capitalism and state‐sanctioned violence. Progress in Human Geography 41(4): 524–533. 10.1177/0309132516646495.

[cag12660-bib-0055] Saik'uz First Nation . 2019. *Saik'uz and Stellat'en First Nations back in court to save the Nechako River and its fisheries: Two First Nations take Rio Tinto Alcan in an effort to stop environmental and cultural damages*. October 16. https://www.saikuz.com/events/newspress-releases/saikuz-stellaten-first-nations-v-rio-tinto-alcan-inc-british-columbia-canada.

[cag12660-bib-0056] Simpson, L. B. 2017. As we have always done: Indigenous freedom through radical resistance. Minneapolis, MN: University of Minnesota Press.

[cag12660-bib-0057] Sloan Morgan, V. , and M. Farrales . 2020. *Youth‐centred performances and events on community, environment, and desires for their future*. Health Arts Research Centre. http://healtharts.ca/youth-centred-performances-and-events-on-community-environment-and-desires-for-their-future/.

[cag12660-bib-0058] Tuck, E. 2009. Suspending damage: A letter to communities. Harvard Educational Review 79(3): 409–427.

[cag12660-bib-0059] Tuck, E. , and K. W. Yang . 2012. Decolonization is not a metaphor. Decolonization: Indigeneity, Education & Society 1(1): 1–40. https://jps.library.utoronto.ca/index.php/des/article/view/18630/15554.

[cag12660-bib-0060] Tuck, E. , K. W. Yang , and R. Gaztambide‐Fernández . 2015. Citation practices challenge. *Critical Ethnic Studies* . http://www.criticalethnicstudiesjournal.org/citation-practices.

[cag12660-bib-0061] UNBC (University of Northern British Columbia) . 2019. UNBC appoints climate change and water security research chair in partnership with NSERC and Rio Tinto. November 4. https://www.unbc.ca/newsroom/unbc-stories/unbc-appoints-climate-change-and-water-security-research-chair-partnership-nserc-and-rio-tinto.

[cag12660-bib-0062] Voosen, P. 2016. The Oaxaca incident: A geographer's efforts to map a Mexican village reveal the risks of military entanglement. Chronicle of Higher Education 62(6): B6–B11.

[cag12660-bib-0063] Voyles, T. B. 2015. Wastelanding: Legacies of uranium mining in Navajo country. Minneapolis, MN: University of Minnesota Press.

[cag12660-bib-0066] Whyte, K. P. 2017. Indigenous climate change studies: Indigenizing futures, decolonizing the Anthropocene. English Language Notes 55(1–2): 153–162.

[cag12660-bib-0067] Whyte, K. P. . 2018. Indigenous science (fiction) for the Anthropocene: Ancestral dystopias and fantasies of climate change crises. Environment and Planning E: Nature and Space 1(1–2): 224–242. 10.1177/2514848618777621.

[cag12660-bib-0064] Wilson, L. 2020. First Nations in B.C. Fighting Rio Tinto to save Nechako River and Fishery. *APTN*, June 25. https://www.aptnnews.ca/featured/first-nations-in-b-c-fighting-rio-tinto-to-save-nechako-river-and-fishery/.

[cag12660-bib-0065] Wood, L. , D. Kamper , and K. Swanson . 2018. Spaces of hope? Youth perspectives on health and wellness in Indigenous communities. Health & Place 50(March): 137–145. 10.1016/j.healthplace.2018.01.010. 29453031

[cag12660-bib-0068] Ybarra, M. 2019. On becoming a Latinx geographies killjoy. *Society + Space* (blog). January 23. https://www.societyandspace.org/articles/on-becoming-a-latinx-geographies-killjoy.

